# Challenges and Advances in Chimeric Antigen Receptor Therapy for Acute Myeloid Leukemia

**DOI:** 10.3390/cancers14030497

**Published:** 2022-01-19

**Authors:** Jennifer Marvin-Peek, Bipin N. Savani, Oluwole O. Olalekan, Bhagirathbhai Dholaria

**Affiliations:** Department of Medicine, Vanderbilt University Medical Center, Nashville, TN 37232, USA; jennifer.marvin.peek@vumc.org (J.M.-P.); Bipin.savani@vumc.org (B.N.S.); olalekan.oluwole@vumc.org (O.O.O.)

**Keywords:** immunotherapy, chimeric antigen receptor, acute myeloid leukemia, bone marrow transplantation

## Abstract

**Simple Summary:**

Chimeric antigen receptor (CAR) therapy has increased treatment options for many patients who have failed standard chemotherapy. So far, CAR therapy has been used more frequently in B-cell mediated cancers due to unique challenges posed by patients with acute myeloid leukemia (AML) and concern for life-threatening side effects. This review discusses both challenges to creating effective and safe CARs for use in AML, as well as recent advances in CAR development both in pre-clinical and human studies. Overall, continued improvement in AML CAR therapy would be of great benefit to a disease that still has a high morbidity and mortality.

**Abstract:**

The advent of chimeric antigen receptor (CAR) T-cell therapy has led to dramatic remission rates in multiple relapsed/refractory hematologic malignancies. While CAR T-cell therapy has been particularly successful as a treatment for B-cell malignancies, effectively treating acute myeloid leukemia (AML) with CARs has posed a larger challenge. AML not only creates an immunosuppressive tumor microenvironment that dampens CAR T-cell responses, but it also lacks many unique tumor-associated antigens, making leukemic-specific targeting difficult. One advantage of CAR T-cell therapy compared to alternative treatment options is the ability to provide prolonged antigen-specific immune effector and surveillance functions. Since many AML CAR targets under investigation including CD33, CD117, and CD123 are also expressed on hematopoietic stem cells, CAR T-cell therapy can lead to severe and potentially lethal myeloablation. Novel strategies to combat these issues include creation of bispecific CARs, CAR T-cell “safety switches”, TCR-like CARs, NK CARs, and universal CARs, but all vary in their ability to provide a sustained remission, and consolidation with an allogeneic hematopoietic cell transplantation (allo-HCT) will be necessary in most cases This review highlights the delicate balance between effectively eliminating AML blasts and leukemic stem cells, while preserving the ability for bone marrow to regenerate. The impact of CAR therapy on treatment landscape of AML and changing scope of allo-HCT is discussed. Continued advances in AML CAR therapy would be of great benefit to a disease that still has high morbidity and mortality.

## 1. Introduction

Over the past decade, the emergence of chimeric antigen receptor (CAR) T-cell therapy has revolutionized how we think about cancer therapeutics and harnessing the immune system to fight cancer. Since the FDA approved tisagenlecleucel (Kymriah^TM^) for pediatric and young adult patients with relapsed/refractory (r/r) B-cell acute lymphoblastic leukemia (ALL) in August 2017, there are now four additional CAR-T cell therapies approved for clinical use. Except for idecabtagene vicleucel (Abecma^TM^), which targets B cell maturation antigen (BCMA) for use in r/r multiple myeloma [1], the remaining FDA-approved CAR-T cell therapies all target CD19 and are approved for treatment of various malignancies of lymphoid lineage including B-ALL, diffuse large B-cell (DLBCL), follicular, and mantle cell lymphoma [2,3,4,5]. 

Despite the increasing availability of CAR T-cell therapy, the success thus far has largely been in treating B-cell and plasma cell driven hematologic malignancies. Developing CAR T-cells for acute myeloid leukemia (AML) has posed some unique challenges limiting their widespread availability. Outcomes for patients with AML treated with standard chemotherapy, while slowly improving, remain poor with an estimated five-year survival of 40–55% in patients less than 60 years old and only 10–15% in those greater than 60 [6], driving the need for new targeted immunotherapies. 

In this review, we discuss barriers to development of AML CARs, potential strategies to circumvent these barriers, and therapeutic targets currently under investigation for AML CARs. 

## 2. Overview of CARs

Chimeric antigen receptors (CARs) are synthetic receptors created from a combination of a ligand-specific extracellular domain (often an antibody-derived single-chain variable fragment (scFv)), a T-cell receptor derived CD3ζ domain, and one or more intracellular co-stimulatory (or activation) domains [7]. While the scFv provides antigen specificity, the co-stimulatory domains are designed to activate the effector cell on which the CAR resides. First generation CAR T-cells possess just a single activation domain (CD3ζ). Due to insufficient persistence and T-cell activation, first generation CARs have been replaced by second and third generation varieties. Second generation CARs use either CD28 or 4–1BB co-stimulatory domains, whereas third generation may incorporate multiple including CD28, 4–1BB, ICOS, and/or OX40 [8]. Fourth generation CAR T-cells, sometimes known as “T-cells redirected for universal cytokine-mediated killing” or “TRUCKs”, are less defined in the literature, but are designed to additionally secrete cytokines into the tumor microenvironment and may co-express additional proteins such as chemokine receptors, switch receptors, and bispecific T-cell engagers (BiTEs) [9,10]. Adoptive transfer of autologous CAR T-cells should result in T-cell activation upon recognition of its target antigen, and subsequent killing of the cancer cell via perforin, granzyme, and recruitment of natural cancer-specific immune responses [11].

An advantage of using antibody-derived scFvs in CAR T-cells is that they function in a major histocompatibility complex (MHC) independent manner [11,12]. However, intracellular antigens are not typically accessible to CARs, as these can only be recognized after being processed and presented by MHC molecules [13]. 

Side effects attributable to CARs are generally related to cytokine release syndrome (CRS) and immune effector cell-associated neurotoxicity syndrome (ICANS) [8,14]. Though anaphylaxis to the murine scFv has been reported, it is not a common event [15]. Expression of the CAR-targeted antigen by other cell types can also lead to on-target/off-tumor toxicity and healthy tissue damage. 

### The Ideal CAR Target in AML

The goal of CAR T-cell therapy is to selectively eliminate cancerous cells while sparing non-cancerous tissue, and to provide sustained anti-cancer immunosurveillance that prolongs remission. 

In AML, relapse is often attributed to leukemic stem cells (LSCs), which are presumed to be chemo-resistant and capable of re-initiating the malignancy [16]. Therefore, many AML patients proceed to allogeneic stem-cell transplant (SCT) with the hope that the graft versus leukemia effect mediated by donor T-cells will eliminate residual LSCs [17]. SCT, however, is not without additional morbidity and mortality, as graft T-cells are not specifically selected for leukemia and may recognize normal tissues in the recipient leading to graft-versus-host disease (GVHD). In comparison, CAR T-cells could be theoretically advantageous in that they could provide that immune surveillance specifically against leukemia. 

The ideal target for an AML CAR should be highly expressed on AML blasts and LSCs, but not expressed on healthy tissues or hematopoietic stems cells (HSCs). Such a target would maximize the immune-mediated anti-leukemic effect while minimizing the potential for off-target side effects. While simple in concept, finding an ideal target for an AML CAR has proven to be much more difficult in practice. 

## 3. Barriers to Development of AML CARs

In 2013, Ritchie and colleagues reported on the first clinical trial for CAR T-cells in relapsed AML using second generation CARs against the Lewis Y antigen [18]. Out of the four patients in the trial, none demonstrated a sustained remission and there was limited expansion of the CAR T-cells. While they were able to show safety and tolerability in AML patients, the study highlighted many of the barriers in development of AML CARs, including lack of AML-specific antigens, antigen escape, the AML-induced immunosuppressive microenvironment, and difficulties with harvesting T-cells from AML patients. 

### 3.1. Lack of AML-Specific Antigens

AML has among the lowest mutational burden compared to other solid organ malignancies, thus, unsurprisingly, AML seems to possess relatively fewer neoantigens that can be targeted by CAR therapy compared to other malignancies [19]. Commonly used AML CAR targets such as CD33 and CD123, which are present in approximately 80–90% and 70–80% of AML patients, respectively, are also expressed on HSCs and on normal myeloid progenitor cells [20,21,22]. This lack of specificity may result in undesired toxicity, prolonged severe myelosuppression, and transfusion dependence. This issue is unique to AML CARs, as CD19 CAR T-cells eliminate solely B-cells which can easily be replaced with IVIg. In addition to being present on HSCs, CD33 is also present on hepatic Kupffer cells, which raises the risk of life threatening veno-occlusive disease which has been observed after treatment with gemtuzumab ozogamicin, a monoclonal antibody-drug conjugate against CD33 [23]. While some AML-specific antigens have been identified such as mutated *NPM1*, *IDH1*, and *IDH2*, these markers are intracellular and therefore not accessible by conventional CARs [13,24,25]. 

Different approaches have been attempted to expand the pool of CAR targets for AML and/or mitigate the severe myelosuppression by limiting the in vivo persistence of the CARs. Altering co-stimulatory domains can affect CAR T-cell persistence, such as using CD28 rather than 4–1BB, may shorten CAR T-cell persistence [26]. The effect of differing co-stimulatory domains is relatively minor, however [27,28,29]. Creation of CARs using mRNA electroporation instead of viral transduction has also been used to prevent long-term persistence of the T-cells as mRNA degradation results in CAR T-cell death. Cummins and colleagues treated six patients with r/r AML with CD123 CAR T-cells created by mRNA electroporation and demonstrated a good safety profile, noting only mild fevers and low-grade CRS [30]. As expected, the CAR T-cells were only transiently detected, but unfortunately there was no measurable anti-tumor response. Electroporation has been hindered by other issues in addition to diminished persistence, including a longer time to manufacture, and lower CAR T-cell viability limiting its clinical utility [31,32]. 

A more effective strategy that is being used to limit the CAR T-cell life span is incorporating a “safety switch” to eliminate the T-cells if there is significant toxicity or life-threatening myelosuppression. One of the initial “safety switches” was created using a HSV-thymidine kinase suicide gene that allows for depletion of the cells upon administration of a prodrug [33]. However, given the long latency from prodrug administration to activation, as well as preventing the use of ganciclovir for treating potentially life-threatening viral infections, this method has largely been replaced. A more popular method is modifying the CAR T-cell to co-express inducible caspase-9 (iCASP9) fused to FK506 binding protein such that, when given AP1903, the molecules dimerize, and the cell undergoes apoptosis [34]. This system has been tested in five pediatric patients who developed GVHD after receiving CD19 CAR T-cells after SCT for r/r ALL. A single dose of AP1903 eliminated more than 90% of the T-cells within 30 min [35]. The iCASP9 system was also incorporated into CLL CAR-T cells for pediatric patients with r/r AML, but did not have to be activated [36]. Other groups are engineering CAR T-cells that co-express a surface antigen for which a monoclonal antibody exists such as EGFR/cetuximab and CD20/rituximab, or have depleted the CAR T-cells using antibodies against endogenous receptors such as CD52 with alemtuzumab [37,38,39,40]. Such safety switches could also help in the event of transduction of leukemic cells and/or high-grade CRS or ICANS [41]. 

Controllable CARs whose function can be reversed is another potential strategy to limit toxicity. Tetracycline (Tet)-ON/Tet-OFF inducible CAR19 T-cells, responsive to a tetracycline analog, have been developed in vitro for B-cell lymphomas [42,43]. Benmebarek and colleagues recently developed a controllable platform utilizing synthetic agonist receptor (SAR) transduced T-cells in combination with AML-targeting tandem svFv constructs [44]. In their system, the SAR T-cells would only be activated in the presence of their CD33 or CD123 scFv construct, but would otherwise remain inert. Their system was tested ex vivo as well as in AML xenograft models. One advantage of these types of reversible CARs over the previously mentioned “safety switches” is that they could be reactivated to induce re-expansion when that is needed e.g., in the setting of waning response or relapse within the life span of the CAR T-cells. 

Another method being to limit CAR therapy duration and minimize toxicity and myelosuppression is engineering CAR NK-cells rather than T-cells. Not only is the risk of on-target/off-tumor toxicity lower due to their limited lifespan, but they are less associated with CRS and ICANS, as CAR NK-cells usually produce a different cytokine profile than CAR T-cells [45]. CAR NK-cells can additionally eliminate cancer cells in a CAR-independent manner through their regular cytotoxic mechanisms which may be helpful in a heterogenous tumor environment. While CAR NK-cells have been studied more in B-cell malignancies than AML, in vitro CAR NK-cells have been shown to robustly kill AML cell lines in vitro [46]. Unfortunately, in the first phase I clinical trial in which three patients with r/r AML were treated with anti-CD33 CAR NK-cells, there was no obvious clinical efficacy [47]. Studies now are investigating the use of stimulatory cytokines such as IL-2, IL-15, and IL-12, as well as monoclonal antibodies to boost NK cell expansion and cytotoxicity [48]. Clinical trials with CAR NK-cells are still ongoing (e.g., NKX101, see Table 1). 

One approach demonstrated by Kim and colleagues [22], is to create an AML-specific antigen by ex vivo deletion of CD33 from normal HSCs prior to allogeneic hematopoietic cell transplantation (allo-HCT). Subsequent treatment with CD33 CAR T-cells would eliminate CD33+ AML blasts and LSCs and spare CD33- HSCs which will repopulate hematopoiesis after allo-HCT. Their model of “making” CD33 specific CAR for AML was successful in both human-murine xenografts as well as macaques, and avoided prolonged myelosuppression. Vor^©^ Biopharma is exploring this strategy by using CRISPER knock-out of various myeloid antigens (CD33, CLL-1, CD123) from HSCs to protect them from CAR T cells targeting same antigens. A first in human, phase 1 study is testing safety of allo-HCT with CD33 deleted HSC followed by treatment with gemtuzumab ozogamicin in AML (NCT04849910). Bispecific CAR T-cells, which recognize two different epitopes, could also be used to enhance specificity for leukemic cells using a split CAR (BissCAR) system. He and colleagues developed CAR T-cells that require both CD13 and T-cell immunoglobulin and mucin domain-containing protein 3 (TIM3) binding for activation [49]. Since CD13 is expressed not only on AML blasts, but also colon epithelial and kidney tubular cells, targeting CD13 alone could lead to excessive toxicity. TIM3, on the other hand, is expressed preferentially on dendritic cells, macrophages, natural killer (NK) cells, and myeloid cells [50]. While almost no known life essential cells express both, He et al. found that 75% of patient derived AML is dual CD13/TIM3 positive [49]. Therefore, with a bispecific split CAR T system, they eradicated AML in xenograft models with reduced toxicity to HSCs and healthy tissues.

Other groups have attempted to utilize the vast array of cancer-specific intracellular antigens to avoid myelosuppression altogether by engineering TCR T-cells. TCR T-cell therapy benefits from an expanded pool of possible targets, but has been limited due to increased difficulty of ex vivo manipulation and risk of creation of mixed dimers of undefined specificity with endogenous TCRs [51]. In mouse models, adoptive transfer of TCR T-cells has led to lethal autoimmunity due to production of self-reactive T-cells [52]. A number of different innovations have been trialed to combat this issue including knockout of the TCR or modification of the TCR by adding on additional amino acids or fusing the signaling components [51,53]. Another alternative is development of TCR-like CAR T-cells which are made from a scFv and CAR signaling machinery, but are instead generated to recognize peptide in the context of MHC class I molecules [54]. TCR CAR T-cells have successfully targeted intracellular synovial sarcoma X breakpoint 2 (SSX2) in the context of HLA*0201 as well as mutated nucleophosmin (NPM1) in the context of HLA-A2 in AML cancer cell lines [55,56]. Xenograft models using TCR CAR T-cells generated against the Wilms’ Tumor Antigen-1 (WT1)/HLA*2402 complex were additionally enhanced by dendritic cell vaccination [57]. In a clinical trial with 12 AML patients, WT-1 TCR CAR T-cells given prophylactically post-SCT improved relapse free survival from 54% in the comparator group to 100% in the treatment group (*p* < 0.001) [17]. However, the generalizability of TCR CAR T-cells to a broad population setting is limited due to their restriction to a specific HLA type.

Despite these options, most clinical trials for AML CAR T-cell therapy are seen as a bridge to transplant, with the goal of eradicating LSCs to reduce relapse rates and avoid life-threatening myelosuppression [13]. While many new targets for AML CARs are under investigation, they are still primarily in the early phase of research of development. Transient CAR T-cell are promising in their avoidance of prolonged myeloablation and cytotoxicity, but also unfortunately lose their benefit of continued anti-cancer immune surveillance and therefore may exhibit reduced clinical efficacy.

### 3.2. Antigen Escape

While not a unique concern with AML CARs, antigen escape caused by down-regulation of the target epitope is the most common cause of relapse seen after anti-C19 CAR T-cell therapy [58]. Creation of a tandem CAR, which is a bispecific CAR that recognizes two epitopes within a single activation domain, may avoid this issue. A tandem CAR activates upon engagement of either one of its receptors, making it harder for tumors to avoid detection [8,59]. CD123-CD33 tandem CAR T-cells have been tested successfully ex vivo in leukemic cells from AML patients, as well as in xenograft models [37]. C-type lectin-like molecule-1 (CLL-1), which is a transmembrane protein expressed on myeloid cells, leukemic blasts, and LSCs, but not HSCs [60,61], has been a popular target for bispecific CARs. CLL1-CD33 CAR T-cells have not only demonstrated potent killing effects on AML cell lines in preclinical studies, but CLL1-CD33 compound (or dual expressing) CAR T-cells also induced remission in a 6-year-old child with r/r AML with complex karyotype [62]. Clinical trials for CLL1-CD33 and CLL1-CD123 CAR-T cells in AML are currently ongoing.

Alternatively, several groups have tried to combine CARS with additional therapies that upregulate the CAR target antigens to overcome the low antigen density caused by AML target downregulation. For example, all-trans retinoic acid (ATRA) induced expression of CD38 and folate receptor-β (FRβ), which in combination with CD38-specific CAR T-cells resulted in elimination of AML blasts in preclinical studies [63,64]. Histone deacetylase inhibitors enhanced natural killer group 2 member D (NKG2D) ligand expression on in vitro AML lines resulting in robust NKG2D-specific CAR activity [65]. Hypomethylating agents (HMAs) such as azacytidine have been shown to up-regulate CD123 expression in xenograft models [66]. Decitabine increased CD19 and enhanced the cytotoxic effect of CD19-specific CAR T-cells in patients with r/r B-ALL [67], but has not yet been tested in combination with AML CARs in clinical trials.

### 3.3. AML-Induced Immunosuppressive Microenvironment

Activity of CARs against AML may also be limited by the immunosuppressive microenvironment that AML creates. AML blasts not only downregulate MHC class I and MHC class II molecules, but they also express inhibitory ligands such as PDL-1, CD276, and galectin-9 [68,69]. T-cells from the plasma of AML patients exhibit decreased proliferation compared to healthy controls, tend to polarize monocytes to an immunosuppressive phenotype, and are more likely to be suppressor Tregs [70,71]. These multiple inhibitory pathways can lead to T-cell exhaustion, dysfunction, and/or poor persistence [72]. An immunosuppressive environment would limit the efficacy of immunomodulatory therapies such as CARs, and studies have indeed found that CAR T-cells from non-responders have up-regulated pathways in apoptosis, likely induced by the AML tumor microenvironment [73].

Limited options are available to combat the anti-inflammatory environment created by AML blasts. In a clinical trial of 14 children with B-ALL, addition of PD-1 blockades to CD19 CAR T-cell therapy improved persistence of the CAR T-cells and 7/14 patients maintained partial or complete remission (CR) [74]. Checkpoint inhibitors are currently being investigated as combination therapy with HMAs for r/r AML, but not yet with AML CARs [72].

### 3.4. Issues with Quality of Autologous Cells

Finally, harvesting T or NK cell product for CAR T manufacture may prove to be difficult due to of prior exposure of intense chemotherapy in most patients with AML [75]. T-cells from AML patients can be particularly difficult to manipulate ex vivo, and are often more expensive than in other hematologic malignancies [76]. Altering the cytokines used for CAR production (e.g., using IL-15 instead of IL-2) has been shown to improve yield, and is currently a strategy employed by at least one recruiting clinical trial (see Table 1) [41].

Another potential solution is using allogeneic T-cells from healthy donors for CAR manufacturing. The main risks of allogeneic CARs are GVHD and poor in vivo expansion and persistence due to alloreactivity [77]. “Universal CARs” generated by genetic deletion of the TCR β-chain are one attempt to circumvent the risk of GVHD [78]. The first study using universal CAR T-cells was against CD19 (UCART-19) in two infants with r/r B-ALL who both achieved remission and underwent successful SCT [79].

Universal CAR T-cells against CD123 with a safety-switch mechanism are currently in clinical trials for use in r/r AML [80]. Based on preliminary results from the first three patients who completed treatment, one patient showed partial remission and two patients achieved CR, although with incomplete hematologic recovery [81]. Side effects included grade 1 CRS that subsided within 48 h. UCART-cells were still detectable six months after dosing in non-transplant patients. Enrollment in a phase 1A study of UCART123 T-cells is ongoing (see Table 1).

Allogeneic NK cells are likewise another therapy under investigation. A clinical trial utilizing allogenic NK cells targeting the NKG2D ligand is currently enrolling (NCT03623944, Table 1). Likewise, universal CAR-NK cells are in phase I trials for B-cell malignancies although none to date are in use for AML [82].

Use of γδ CAR T-cells instead of the conventional αβ T-cells may be another strategy to mitigate GVHD risk associated with allogenic CARs. γδ T-cells are part of the innate lymphocyte family and do not possess CD4 or CD8 [83]. They are not alloreactive and therefore not capable of causing a GVH response. Nonetheless, upon activation they are cytotoxic and can function as professional antigen presenting cells. γδ CAR T-cells have been shown to be effective against CD19+ leukemia cells lines in vitro [84]. However, isolation of γδ CAR T-cells is frequently limited by low circulating numbers for harvest, as well as increased difficulty with expansion ex vivo [84]. An observational trial investigating the feasibility of isolating γδ T-cells from patients with AML and inserting in CARs just closed in March 2021 (NCT03885076). Results are awaited.

## 4. Experimental Targets under Investigation for AML CARs

**Figure 1:**
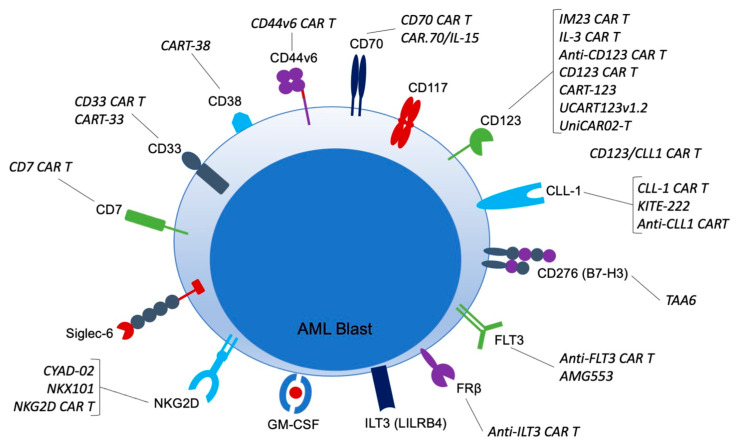
CAR targets in AML and CAR therapies currently in clinical trials. CAR = chimeric antigen receptor; AML = acute myeloid leukemia; CD = cluster of differentiation; CLL-1 = C-type lectin-like molecule-1; FLT3 = FMS related receptor tyrosine kinase 3; FRb = folate receptor b; HSC = hematopoietic stem cell; GM-CSF = granulocyte-macrophage colony-stimulated factor; ILT3 = Ig-like transcript 3; LSC = leukemic stem cell; NKG2D = natural killer group 2 member D; Siglec-6 = sialic acid binding Ig-like lectin 6.

There are over 10 different targets for AML CARs currently in clinical trials (Table 1; Figure 1).

Numerous others are being investigated in preclinical models, many of which anticipate moving into clinical trials soon (Table 2). Each target possesses a unique distribution on immune cells and healthy tissues. Not all possible targets for AML CARs can be discussed in this review article, but some of the most promising are discussed below.

### 4.1. CD7

CD7 is transmembrane protein estimated to be expressed by 30% of adult AML patients [11]. When present, it is typically associated with a more aggressive disease course and resistance to chemotherapy [85]. It is classically found on activated T-cells, but is also found on NK cells and some lymphoid and myeloid progenitors. Its function is thought to be redundant, as CD7 deficient mice retain normal T-cell function. Since CD7 is expressed on activated T-cells, it must be genetically removed from the CARs prior to transfer [86]. The Gomes-Silva group published the first preclinical data demonstrating that CD7 CAR T-cells protected against severe leukemia on xenograft models [87]. Clinical trials using CD7 CARs are currently ongoing (Table 1).

### 4.2. CD33

CD33 is a sialic acid binding immunoglobulin that is expressed primarily on myeloid lineage cells, including myeloid progenitor cells [88]. It is additionally expressed on neutrophils, NK cells, B-cells subset, and Kupffer cells in the liver [89]. Approximately 85–90% of AML cases express CD33, and it is also expressed on LSCs making it a popular antigen for CAR T-cell therapy [90]. The first patient who received CD33 CAR T-therapy was a 41-year-old with r/r AML who initially had marked disease regression, but soon relapsed [91]. Another single-center phase I clinical trial designed to evaluate the feasibility and safety of CD33 CAR-T cells in r/r AML enrolled 10 patients, three of which received cells [92]. Many side effects were noted, including CRS, ICANS, tumor lysis syndrome (TLS), grade 3 respiratory distress syndrome, and septic shock. All three died from disease progression. Notably, however, there was no reported hepatotoxicity in these trials despite CD33 expression on normal healthy Kupffer cells. Clinical trials investigating CD33 CARs as well as bispecific CD33-CLL CARs are currently recruiting (Table 1).

### 4.3. CD38

CD38 is known for its expression on plasma cells [1] However, it is also expressed by other lymphoid cells as occasionally myeloid cells. In total, six patients with r/r AML post-allogenic SCT were treated with CD38 CARs and four patients achieved CR, with 50% of them relapsing at six months [93] There are no current clinical trials utilizing CD38 CARs for AML in the United States.

### 4.4. CD44v6

CD44 is an adhesive receptor that is expressed broadly on multiple tissue types. The splice site variant, CD44v6 is present in up to 60% of AML cases and is relatively tumor-restricted, although expressed in low levels on normal cells including T-cells, monocytes, and keratinocytes [94]. In preclinical studies, CD44v6 CAR T-cells had potent effects on primary AML cells while sparing normal HSCs [94]. The results were consistent in xenograft mouse models and now CD44v6 is being used as a target in a clinical trial in Italy.

### 4.5. CD70

CD70 or the tumor necrosis factor receptor ligand is upregulated on immune cells when activated, but is otherwise not expressed on hematopoietic cells [95]. When expressed, CD70 correlates with poor survival. Targeting CD70 with a monoclonal antibody (cusatuzmab) successfully eliminated AML stem cells when used in combination with HMAs, making it an intriguing target for CAR therapy [95]. In preclinical studies, CD70 CARs demonstrated cytolytic activity against AML blasts and LSCs, but not HSCs [96]. Currently there is one clinical trial recruiting using CD70 CAR-T cells and another using CD70 NK cells transduced with IL-15 for use in r/r AML (Table 1).

### 4.6. CD117

CD117, otherwise known as c-kit, is the cognate receptor for stem cell factor and plays a critical role in HSC homeostasis [97]. It is expressed on 80–90% of AML blasts and is related to adverse clinical outcomes [98]. In xenograft models, anti-CD117 CAR T-cells completely eradicated both healthy and leukemia disease [97]. The CAR T-cells were then depleted with ATG and rituximab, and hematopoiesis was rescued by SCT. Given the high expression of CD117 on HSCs, CAR therapy against CD117 would be restricted to use as a bridge to transplant. Studies on CD117 and AML are only in the preclinical phase at this time.

### 4.7. CD123

CD123 or interleukin-3 receptor subunit alpha is positive in 70–80% of AML patients, and is expressed predominantly on myeloid lineage cells [21]. CD123 positivity is associated with increased risk of treatment failure [99]. Out of all AML CAR targets, CD123 currently has the most ongoing clinical trials including universal CD123 CARs, bispecific CARs, and CARs with safety switches (Table 1). Anti-CD123 CAR T-cells have successfully eliminated leukemic blasts in multiple pre-clinical studies [66,100,101]. In the first clinical trial of six patients with r/r AML, CD123 CAR T-cells brought 3/6 to CR, 66% of which underwent subsequent allogeneic SCT [102].

### 4.8. CD276 (B7-H3)

CD276 is a transmembrane protein for which overexpression has been associated with a variety of human cancers, including AML blasts (especially with the monocytic subtype) [103]. It is present in 39–80% of bone marrow specimens from patients with AML. All published data thus far using CARs against CD276 is preclinical, but shows promising efficacy in xenograft models and has even been couple in a tandem car with CD70 [103,104,105].

### 4.9. CLL-1

CLL-1, otherwise known as C-type-lectin-like molecule 1, is expressed on >80% of AML blasts [20,106,107]. It is becoming an increasing popular CAR target for AML as it is highly expressed on chemotherapy resistant LSCs, but it notably absent on granulocyte progenitors and non-hematologic tissues [108]. Multiple preclinical models support strong anti-leukemic activity of CLL-1 directed CARs without destruction of normal HSCs [109,110]. The first patient treated with CLL-1 CAR T-cells published by Zhang and colleagues remained in CR for >10 months [111]. His course was complicated only by self-resolving grade I-II CRS. In a small clinical trial with four pediatric patients with r/r AML, CLL CAR T-cells with the iCASP safety switch, 75% achieved CR with only reported low grade adverse events not requiring activation of the safety switch [36]. Several singular and tandem anti-CLL-1 CAR clinical trials are ongoing (Table 1).

### 4.10. FLT3

Fms-related receptor tyrosine kinase 3 (FLT3)’s role is to maintain survival of normal HSCs [20]. The FLT3-ITD mutation is present in 24% of patients with AML while the FLT3-TKD mutation is present in ~5%. Both are associated with a poor prognosis [112,113]. Multiple inhibitors of FLT3 are FDA approved for use in FLT3^+^ AML including gilteritinib, midosaurin, and quizartinib, so it is not surprising that FLT3 CARs are being investigated. Currently all published work is preclinical, but has showed promising activity against leukemic cell lines [114,115,116,117,118]. Many groups are using safety switches to allow for bone marrow recovery afterwards, given that FLT3 also depleted HSCs [38]. Several clinical trials including one in the United States is actively recruiting.

### 4.11. FRβ

The folate receptor (FR) family is a group of four folate-binding protein receptors. FRα is expressed on epithelial cells and has been used as a target for CAR-T cells in ovarian cancer [119]. FRβ is a myeloid lineage antigen that is upregulated in the setting of malignancy, and expressed in up to 70% of AMLs [120]. FRβ-specific CARs demonstrated anti-leukemic effect in vitro and in vivo with sparing of the HSC colony [64,121]. To date, no FRβ CARs have been used in humans.

### 4.12. GM-CSF (CD116/CD131)

CAR T-cells are also being investigated on preclinical trials against granulocyte-macrophage colony-stimulated factor (GM-CSF) or CD116/CD131 [122]. CD116 is overexpressed on 63–78% of AML cases, especially when FLT3 mutated [123,124]. In xenograft models, anti-GM-CSF CAR-T cells had potent anti-tumor effects. More studies are needed to determine clinical utility.

### 4.13. ILT3 (LILRB4)

Leukocyte immunoglobulin-like receptor B (LILRB4 or ILT3) is expressed on myeloid antigen presenting cells and suppresses T-cell activation and proliferation [125]. Increased ILT3 expression in AML cells is thought to represent an attempt to avoid immune surveillance, especially of the myeloid subtype [126]. CAR T-cells against the receptor show activity against monocyte AML cells with sparing of healthy HSCs [127]. The first clinical studies are ongoing (Table 1).

### 4.14. NKG2D

Natural killer group 2 member D (NKG2D) ligands are a promising target that is expressed only on healthy NK cells, γδ T-cells, CD8^+^ T-cells, and some subsets of CD4^+^ T-cells. NKG2D is upregulated in response to DNA damage, inflammation, and malignant transformation, and is therefore detectable on a wide number of malignancies including AML, albeit in low frequency [47,128]. Even low-level ligand expression resulted in robust CAR activity in in vitro models [65]. HDAC inhibition also enhanced NKG2D expression and CAR T-cell mediated elimination [129]. In a phase I clinical trial of NKG2D CAR T-cells in patients with AML, myelodysplastic syndrome (MDS), and multiple myeloma without lymphodepleting conditions, they found that expansion and persistence of the CAR T-cells was limited, but functional activity was detected [130]. No dose limiting toxicity and/or CRS, ICANs was observed. With the single dose of NKG2D CAR T-cells given, they found no objective anti-tumor response. Currently, multiple new clinical trials involving NKG2D CAR NK and CAR T-cells are ongoing (Table 1).

### 4.15. Siglec-6

Sialic acid binding immunoglobulin like lectin 6 or Siglec-6 is an inhibitory molecule expressed on immune cells and the placenta that is also commonly expressed in leukemias, including approxiamtely 60% of AML blasts and stem cells [131]. It is currently being evaluated in many preclinical trials for leukemias and lymphomas, and has shown to induce CR in xenograft AML models with sparing of HSCs [131].

### 4.16. TIM3

The T-cell immunoglobulin and mucin domain-3 (TIM3) plays a role in regulation of inflammation by regulating macrophage activation, inhibits Th1 and Th17 responses, and attenuates TCR signaling [20]. One of the mechanisms by which AML shields itself from the immune system is to produce TIM3 to reduce cytotoxic killing by the immune system. TIM3 is only found in 6% of patients with AML [132], but when present it is highly expressed and associated with poor prognosis. In 2020, Brunner and colleagues published data from their phase 1 clinical trial using a monoclonal antibody inhibitor of TIM3 (sabatolimab) in combination with an HMA in high-risk MDS and AML [133]. They found a 12-month progression free survival rate of 44% in the 34 patients enrolled with newly diagnosed AML. While there are no current clinical trials utilizing anti-TIM3 CAR-T cells, they have been effective in in vitro and in vivo xenograft models [134].

## 5. Potential Impact of CAR on the AML Treatment Landscape

### 5.1. Target Population for AML CARs

As the technology for CARs continues to expand, we anticipate that CARs will become more widely available for patients with AML. Currently, to be eligible for most clinical trials, patients must have r/r disease and be ineligible for intensive salvage chemotherapy or have relapsed post-SCT (Table 1). When trying to decide which patients would benefit the most from CAR in addition to the r/r population, we can again look to studies in ALL treated with commercially available CD19 CAR T-cell therapy. Lu et al., reported that in their cohort of 14 patients with chemotherapy-refractory minimal residual disease (MRD-positive) B-ALL, one cycle of CD19 CAR T-cell therapy put 12 of the 14 into MRD-negative remission with a two-year event-free survival (EFS) of 61.2% and an overall survival (OS) of 78.6% [135]. For patients with AML who plan to undergo SCT, MRD status is a reliable indicator of post-allogenic SCT success with two-year overall survival rate of 66.8% in patients with MRD-negative diseases versus 30% in MRD-positive disease [136,137]. Therefore, CAR therapy may a valuable role for MRD-positive patients planning on future SCT. CNS disease may be another area where CAR therapies have increased efficacy. In a post-hoc analysis of 195 patients with r/r CD19^+^ ALL with and without CNS disease who received CD19 CAR T-cells, the same proportion of patients achieved CR at 28 days after infusion [138]. Additionally, CRS and ICANs rate were identical across those with and without CNS disease. In a population that classically is high risk with high rates of relapse, these results are promising and drive the need for studies examining CAR therapies in those with CNS AML.

### 5.2. Bridging Treatment for AML CAR

Optimization of the AML CARs during the “bridge period” is another area of needed study. On average, it takes two to six weeks from T-cell apheresis to CAR infusion as time [139]. Many clinic trials have a high drop-out rate due to this “bridge period” as patients can have progression of disease or suffer from disease-related complications. In a review of 62 adults with B-ALL, of the 12 patients who received bridging or cytoreductive chemotherapy prior to CAR T-cell infusion, the median OS was 16.3 months compared to 4.3 months in those who did not receive bridging therapy (*p* = 0.04) [139]. Interestingly, when comparing high or low intensity bridging chemotherapy, there was no improvement in OS in the group that received the high intensity regimen even in the high disease burden subgroup. High intensity bridging chemotherapy was, however, associated with more infectious complications and therapy related toxicity. If these results were to be consistent for patients with AML, a reduced intensity bridging chemotherapy regimen may preserve CAR T-cell candidacy in patients with rapidly progressing disease.

### 5.3. Combining Targeted Inhibitors with CAR Products

Combination of CAR therapy with commercially available small molecule inhibitors (e.g., *HDAC*, *FLT3*, *IDH2*, *BCL2* inhibition) for AML is another area of promise and exploration. As previously mentioned, selective HDAC inhibition increased the expression of NKG2D and enhanced CAR NK-cell efficacy [65]. FLT3 inhibitors used on combination with FLT3 CAR T-cells lead to improved elimination of AML blasts in vitro and in vivo [114]. Venetoclax, a BCL-2 inhibitor, was recently demonstrated to directly activate T-cells and increase their cytotoxicity against AML in cell lines and mouse models via reactive oxygen species formation [140]. In a B-ALL tumor cell line, venetoclax prior to CAR T-cell therapy enhanced the cytotoxic effect by upregulation of CD19 expression and pro-apoptotic proteins [141]. DNA methyltransferase inhibitors, such as azacitadine and decitabine, have also enhanced the anti-leukemic effect of CD123-directed CAR T-cells in preclinical models [54,142]. As CAR T-cell therapy for AML develops, synergistic effects between small molecule targeted inhibitors and CAR therapies may be further elucidated.

### 5.4. Integrating AML CARs with Allogenic SCTs

The increasing availability of AML CARs will likely alter how we think about allogenic SCTs, which is currently the standard of care for patients with intermediate or unfavorable risk AML in CR1 [143]. Data again gathered from our experience with CAR T-cell therapy in B-ALL sheds light on who may or may not benefit from consolidative SCT for AML after CAR therapy. In a landmark trial by Park and colleagues using CD19 CAR T-cells in B-ALL, 83% of the study subjects achieved CR with 67% achieving MRD negativity. Out of the MRD-negative patients, 1/3 underwent consolidative SCT with 35% later relapsing. Interestingly, there was no difference in event free survival (EFS) among MRD-negative patients who did or did not undergo SCT [144]. This suggests that performing SCTs in patients with MRD negative disease after CAR T-cell therapy may not be beneficial. However, we know that better remissions before allogenic SCT is indicative of prolonged survival after allogenic SCT, which makes matters more complicated especially in high-risk disease [145,146]. In a clinical trial of 58 patients with r/r B-ALL who had failed targeted CD19 therapy and were given a CD22 CAR T-cell infusion, those who achieved CR (70%) and underwent SCT had a greater EFS and OS than those who did not undergo SCT [147]. High disease burden is also a consistent marker for increased risk of relapse [144,148]. Based on the outcomes of 185 patients who received tisagenlecleucel (Kymriah^TM^), the Pediatric Real-World CAR Consortium reported that high disease burden (defined at >5% marrow or peripheral blasts, CNS disease, or extramedullary disease) was associated with lower CR rates and poorer EFS and overall survival [149]. Specifically, they reported that low disease burden had a one-year EFS of 69% compared to non-detectable disease (72%) and high burden disease (34%), classified at time of CAR T-cell infusion. Overall, we must way the risks of relapse after CAR T-cell therapy for AML versus non-relapse mortality from allogeneic SCT. Ideally, giving patients the best possible response before SCT which may include CAR T-cell therapy should improve outcomes given known poor outcomes with persistent disease.

If the plan is to do a consolidative or tandem SCT, timing is critical. While most patients achieve CR within one month of CAR T-cell infusion, most relapses also occur within the first six-month window [146]. Therefore, ideally, the SCT should be performed within the first three to six months after CAR-T cell infusion after CAR T associated side effects such as CRS/ICANs have resolved. HLA typing and preliminary donor searches should then be done in parallel with evaluation for CAR T-cell therapy.

Finally, CAR T-cell therapy opens the door for using donor-derived CARs post-SCT rather than donor lymphocyte infusions (DLI). In post-SCT relapsed B-ALL, donor derived CAR T-cell infusions have consistently been shown to be superior to DLI, despite ongoing immunosuppressive therapy [150,151,152]. In fact, in patients with high-risk disease, preemptive donor CAR T-infusions post-SCT were shown to be safe, well-tolerated, and had a CR rate of 48% at a median of 5.2 months after infusion [153]. This may, in part, be mediated by the fact that CAR T-cells are more specific for the leukemic blasts and stem cells than a generalized population of T-cells from the donor. Interestingly, Yao and colleagues developed a regimen where donor-derived CAR T-cells specific for CD123 were used as part of a conditioning regimen for a haploidentical SCT in a patient with AML who relapsed after initial SCT and was resistant to multiagent chemotherapy and DLI. Within two weeks of the infusion and SCT, he achieved full donor chimerism and CR with incomplete blood count recovery [154].

Overall, AML CARs have the potential to greatly change the landscape of AML treatment and how we utilize SCT and donor T-cell infusions as tools to achieve longer remissions in patients with high-risk disease.

## 6. Conclusions

Despite advances in therapy for acute myeloid leukemia, outcomes overall continue to be poor. SCT remains the only potentially curative therapy for patients with AML, but is not without significant morbidity and mortality and still carriers a risk of relapse. With the success of CAR T-cell therapy in pediatric relapsed/refractory B-ALL and other B-cell mediated lymphomas, clinical trials for CARs in AML have skyrocketed. Harnessing the immune system for tumor surveillance and to seek out chemo-resistant leukemic stem cells remains the goal of such therapy. However, as discussed in this review, AML has provided several unique challenges for CAR development. The majority of antigens expressed by AML blasts are shared with healthy myeloid progenitors and hematopoietic stem cells leading to prolonged, and sometimes lethal, myeloablation during CAR T-cell therapy. Furthermore, AML creates an immunosuppressive tumor microenvironment that renders CARs less effective than in other malignancies. CARs from AML patients have also been particularly difficult to harvest and manipulate ex vivo owing to the intense chemotherapy that many patients with AML have received. More recent studies and clinical trials are utilizing different strategies to combat these issues, such as creation of bispecific CARs, suicide genes or safety switches, TCR-like CARs, γδ CARs, NK CARs, and generation of universal or allogeneic CARs. Achieving robust anti-tumor response and long-term immunosurveillance must be tightly balanced with avoiding serious prolonged immunosuppression and transfusion dependency with many of the existing AML CAR targets. Novel CAR therapies discussed show significant promise in reducing prolonged myeloablation while retaining efficacy. As CAR technology evolves, hopefully the repertoire of therapeutic options for patients with AML will continue to grow.

## Figures and Tables

**Table 1:** Ongoing CAR therapy trials in AML.

Target Antigen	Name of Drug	CAR Cell Type	Phase	https://clinicaltrials.gov/ (accessed on 21 December 2021) ID	Age (years)	Country
CD7	CD7 CAR-T	T	I/II	NCT04762485	12 to 65	China
CD7	CD7 CAR-T	T	I/II	NCT04033302	Up to 75	China
CD19	CAR-T CD19	T	II/III	NCT04257175	18 and up	Israel
CD33	CD33 CAR-T	T	I/II	NCT04835519	1 to 70	China
CD33	CD33CART	T	I/II	NCT03971799	1 to 35	USA
CD33	CART-33	T	I	NCT02799680	50 and up	China
CD33	PRGN-3006	T	I	NCT03927261	18 and up	USA
CD33/CLL1	Dual CD33-CLL1 CAR-T	T	I	NCT05016063	18 to 70	China
CD38	CART-38	T	I/II	NCT04351022	6 to 65	China
CD44v6	CD44v6 CAR-T	T	I/II	NCT04097301	1 to 75	Italy
CD70	CD70 CAR-T	T	I	NCT04662294	All	China
CD70	CAR.70/IL-15	NK	I/II	NCT05092451	18 and up	USA
CD123	IM23 CAR-T	T	I	NCT03585517	3 to 80	China
CD123	IL-3 CAR-T	T	I	NCT04599543	All	China
CD123	Anti-CD123 CAR-T	T	I	NCT04014881	18 to 70	China
CD123	CD123-CAR-T	T	I	NCT04318678	Up to 21	USA
CD123	CART-123	T	I/II	NCT03556982	14 to 75	China
CD123	CD123 CAR-T	T	I/II	NCT04272125	3 to 75	China
CD123	CD123 CAR-T	T	I/II	NCT04265963	2 to 75	China
CD123	UCART123v1.2	T	I	NCT03190278	18 to 65	USA
CD123	UniCAR02-T	T	I	NCT04230265	18 and up	Germany
CD123	CD123-CAR-CD28-CD3z-EGFRt	T	I	NCT02159495	12 and up	USA
CD123	CART123	T	I	NCT04678336	1 to 29	USA
CD123	CART123	T	I	NCT03766126	18 and up	USA
CD123/CLL-1	CD123/CLL1 CAR-T	T	II/III	NCT03631576	Up to 70	China
CD276	TAA6	T	I	NCT04692948	18 to 70	China
CLL-1	CLL-1 CAR-T	T	I	NCT04219163	Up to 75	USA
CLL-1	KITE-222	T	I	NCT04789408	18 and up	USA
CLL1	Anti-CLL1 CART	T	I/II	NCT04884984	6 to 65	China
CLL1	Anti-CLL1 CART	T	I	NCT04923919	2 to 75	China
FLT3	Anti-FLT3 CAR-T	T	I/II	NCT05023707	16 to 65	China
FLT3	AMG553	T	I	NCT03904069	12 and up	USA
FLT3	TAA05	T		NCT05017883	18 to 70	China
ILT3	Anti-ILT3 CAR-T	T	I	NCT04803929	18 to 70	China
NKG2D	CYAD-02	NK	I	NCT04167696	18 and up	USA
NKG2D	NKX101	NK	I	NCT04623944	18 and up	USA
NKG2D	NKG2D CAR-T	NK	I	NCT04658004	3 to 70	China

CAR = chimeric antigen receptor; AML = acute myeloid leukemia; CD = cluster of differentiation; CLL-1 = C-type lectin-like molecule-1; FLT3 = FMS related receptor tyrosine kinase 3; ILT3 = Ig-like transcript 3; NKG2D = natural killer group 2 member D.

**Table 2:** Experimental Targets in AML CARs.

Target Name	Normal Tissue Distribution	Expression on LSCs	Phase of Development
CD7	activated T-cells, NK cells, lymphoid and myeloid progenitors	Yes	Phase I/II clinical trial
CD33	myeloid cells, myeloid progenitors, Kupffer cells	Yes	Phase I/II clinical trial
CD38	plasma cells, NK cells, B-cells, HSCs (low)	No	Phase I/II clinical trial
CD44v6	activated T-cells, monocytes, keratinocytes	No	Phase I/II clinical trial
CD70	dendritic cells, B-cells	Yes	Phase I/II clinical trial
CD117	HSCs, myeloid progenitors, erythroid progenitors	No	Preclinical
CD123	myeloid cells, myeloid progenitors	Yes	Phase II/III clinical trial
CD276 (B7-H3)	Antigen presenting cells, HSCs (low)	No	Phase I clinical trial
CLL-1	myeloid cells, myeloid progenitors	Yes	Phase I/II clinical trial
FLT3	HSCs	No	Phase I clinical trial
FRb	myeloid cells, HSCs (low)	No	Preclinical
GM-CSF (CD116)	myeloid cells	No	Preclinical
ILT3 (LILRB4)	myeloid antigen presenting cells	No	Phase I/II clinical trial
NKG2D	NK cells, γδ T-cells, CD8^+^ T-cells	No	Phase I/II clinical trial
Siglec-6	B-cells, mast cells, placenta	Yes	Preclinical
TIM3	T-cells, myeloid cells, NK cells	Yes	Preclinical

CAR = chimeric antigen receptor; AML = acute myeloid leukemia; CD = cluster of differentiation; CLL-1 = C-type lectin-like molecule-1; FLT3 = FMS related receptor tyrosine kinase 3; FRb = folate receptor b; HSC = hematopoietic stem cell; GM-CSF = granulocyte-macrophage colony-stimulated factor; ILT3 = Ig-like transcript 3; LSC = leukemic stem cell; NKG2D = natural killer group 2 member D; TIM3 = T-cell immunoglobulin and mucin domain-containing protein 3; Siglec-6 = sialic acid binding Ig-like lectin 6.
